# Smoking Patterns and Stimulus Control in Intermittent and Daily Smokers

**DOI:** 10.1371/journal.pone.0089911

**Published:** 2014-03-05

**Authors:** Saul Shiffman, Michael S. Dunbar, Xiaoxue Li, Sarah M. Scholl, Hilary A. Tindle, Stewart J. Anderson, Stuart G. Ferguson

**Affiliations:** 1 Department of Psychology, University of Pittsburgh, Pittsburgh, Pennsylvania, United States of America; 2 Department of Biostatistics, University of Pittsburgh, Pittsburgh, Pennsylvania, United States of America; 3 Division of General Internal Medicine, University of Pittsburgh, Pittsburgh, Pennsylvania, United States of America; 4 School of Medicine, University of Tasmania, Hobart, Tasmania, Australia; Baylor College of Medicine, United States of America

## Abstract

Intermittent smokers (ITS) – who smoke less than daily – comprise an increasing proportion of adult smokers. Their smoking patterns challenge theoretical models of smoking motivation, which emphasize regular and frequent smoking to maintain nicotine levels and avoid withdrawal, but yet have gone largely unexamined. We characterized smoking patterns among 212 ITS (smoking 4–27 days per month) compared to 194 daily smokers (DS; smoking 5–30 cigarettes daily) who monitored situational antecedents of smoking using ecological momentary assessment. Subjects recorded each cigarette on an electronic diary, and situational variables were assessed in a random subset (*n* = 21,539 smoking episodes); parallel assessments were obtained by beeping subjects at random when they were not smoking (*n* = 26,930 non-smoking occasions). Compared to DS, ITS' smoking was more strongly associated with being away from home, being in a bar, drinking alcohol, socializing, being with friends and acquaintances, and when others were smoking. Mood had only modest effects in either group. DS' and ITS' smoking were substantially and equally suppressed by smoking restrictions, although ITS more often cited self-imposed restrictions. ITS' smoking was consistently more associated with environmental cues and contexts, especially those associated with positive or “indulgent” smoking situations. Stimulus control may be an important influence in maintaining smoking and making quitting difficult among ITS.

## Introduction

Nicotine dependence is considered the primary determinant of persistent cigarette smoking, with individuals typically smoking frequently throughout the day, every day. This serves to prevent nicotine levels from sinking below a point that may lead to the onset of withdrawal [1], [2]. Maintaining nicotine levels above a threshold requires frequent and regular smoking to overcome the rapid clearance of nicotine from the body (half-life of 2–3 hours; [3]), a pattern Russell [4] referred to as “trough maintenance.” Trough maintenance seems to account for the behavior of daily smokers, who smoke frequently and at regular intervals over the course of the day [5].

While daily smoking is the typical pattern in mature smokers, non-daily smoking, which does not support nicotine maintenance, is quite common among young adults [6], [7], many of whom identify themselves as “social smokers” who may smoke mostly with others and for social reasons [8], [9], [10]. Such early patterns are often seen as a transient developmental stage in smoking careers, as continued exposure leads to progression to daily and heavier smoking [11], [12]. However, non-daily smoking is increasingly seen as an established pattern even in smokers beyond the young adult years [8], [13], [14], [15]. Nearly a third of adult US smokers do not smoke daily [16], [17], [18] (though see [6], [7]). Most non-daily smokers (also called intermittent smokers [ITS]) maintain that status over a 1-year period [19]. In a recent study of ITS [15], we found that these adult ITS (three quarters were over 25) had been smoking an average of 19 years, over which time they had consumed more than 40,000 cigarettes, so they are well beyond any initial experimental or developmental period. Despite this long smoking history, these ITS reported smoking an average of only 4 days per week, consuming about 4 cigarettes per day on the days that they smoked. These ITS do not smoke often enough to maintain effective nicotine levels (to be “trough maintainers” in Russell's [4] parlance); they might represent what Russell [4] called “peak-seekers” – smokers who smoke in order to get the positively reinforcing acute effects of smoking, rather than avoid the aversive effects of nicotine withdrawal. Indeed, ITS do not behave as though they are avoiding withdrawal.

Over a recording period of about two months, ITS abstained voluntarily for periods averaging five consecutive days [15], demonstrating a tolerance for nicotine abstinence [20]. In light of this, it is surprising that ITS have substantial difficulty quitting, demonstrating failure rates of 78% – only modestly lower than those seen in DS [21] (see also [22]).

In the absence of a biological imperative to regulate nicotine levels, which is the foundation of nicotine dependence in most models, what might help explain ITS' persistent smoking and their difficulty quitting? One factor might be stimulus control. It's been shown, for example, that exposure to smoking cues, including seeing others smoke, can cue craving and smoking in both young adult ITS [23] and in established adult daily smokers [24], [25]. Smoking is also influenced by a broader range of situational cues such as alcohol exposure or certain physical settings that may come to be associated with it [26]. Thus, if ITS' smoking is substantially associated with certain situational cues, which may serve as discriminant stimuli for acute nicotine reinforcement, exposure to such cues might promote continued smoking and make abstinence difficult in the face of cue exposure.

Tobacco control policies such as environmental smoking restrictions can shape the stimuli associated with smoking and promote the development of stimulus control. By putting pressure on smoking, they may also reduce smoking overall, perhaps eliminating some cigarettes, leaving those that are most compelling for the smoker. Further, pervasive smoking restrictions essentially create distinct smoking and non-smoking environments, strengthening stimulus control. In this context, it is striking that the proportion of adult smokers who are ITS is highest in the states with the most aggressive tobacco control policies [14].

Identifying the particular situational correlates of smoking patterns can also provide clues to smoking motives. For example, if ITS smoked primarily when others were smoking, this may suggest their motives were primarily social. Indeed, non-daily patterns of smoking are often linked to “social smoking” [8], [9], [10], [27], which has important motivational implications: if ITS do not smoke when alone, it would imply that their smoking is motivated by extrinsic social motives, rather than by pharmacological motives, or by any other intrinsically reinforcing aspects of smoking. Conversely, smoking alone would indicate that smoking is being reinforced by motives or stimuli other than social ones. Similarly, examining smoking patterns (e.g., the rate of smoking by time of day) can also help to shed light on smoking motives (e.g., whether ITS are smoking at a constant rate over the course of the day, as would be expected among “trough maintainers,” or whether the timing of their smoking is more varied, suggesting reactivity to environmental cues).

Associations of ITS' smoking with negative affect could also prove important. Baker, Morse, Sherman, & Rivers [28] and others [29],[30] have suggested that affect management is a major motive for smoking. DS typically report smoking to relieve stress as one of their strongest motives [12], [29]. Suprisingly, on questionnaires, ITS were actually more likely than DS to cite stress or anger among their most common smoking situations [15], suggesting that ITS may not just smoke to meet social needs, but may be using smoking to reduce, or cope with, emotional distress. Negative-affect smoking has usually been attributed to smoking to relieve nicotine withdrawal [31], but it has also been suggested that nicotine relieves ordinary emotional distress due to other factors [32]. Conversely, research suggests that nicotine enhances the reinforcement value of experiences that are already reinforcing [33], suggesting that smoking may accompany positive affect. It has also been suggested that some smokers smoke in order to manage levels of arousal [34], so it is also important to examine arousal as an influence in smoking. Such effects would be highly reinforcing, and could help explain ITS' smoking. Thus, shedding light on situational contexts of ITS' smoking – and comparing them to those associated with DS' smoking – may serve to explain differences in what motivates and maintains the two groups' disparate smoking behavior.

Questionnaire measures are of limited value in establishing situational or affective linkages to smoking; they are fraught with psychometric problems, and do not seem to accurately reflect actual smoking patterns [35], [36], [37]. Laboratory cue reactivity studies are another method for examining these associations. Such studies have documented responses to smoking cues not only in increased craving [25], [38], [39], [40], but also in objectively-measured changes in brain activity [41], [42], [43], [44]. A recent cue reactivity study [45] found that ITS and DS reacted similarly to a range of cues, with both groups increasing craving – but not smoking – in response to smoking or alcohol cues, and decreasing craving in response to positive affect cues. This would seem to suggest little difference between DS and ITS in smoking patterns or stimulus control. However, it is not known how well laboratory cue reactivity responses relate to real-world smoking patterns.

To assess real-world smoking patterns, the present study uses Ecological Momentary Assessment (EMA) [46], [47] – collection of real-time, real-world data on multiple occasions – to compare antecedents of smoking and non-smoking occasions [48], in order to characterize the influence of situations and internal states on smoking among adult DS and ITS. This method has been used to study situational associations with smoking in a variety of populations [24], [49], [50], [51].

## Methods

### Subjects

Subjects were 212 ITS and 194 DS recruited for this study via advertisement and promotion. The sample largely overlaps with that reported in several analyses of non-EMA data [15], [45], [52], [53], [54]. To be eligible, all volunteers had to be at least 21 years old, report smoking for at least 3 years, smoking at their current rate for at least 3 months, and not be planning to quit within the next month. DS had to report smoking every day, averaging 5 to 30 cigarettes per day. ITS had to report smoking 4 to 27 days per month, with no restrictions on number of cigarettes. By design, we oversampled African-American (AA) smokers, because national surveys indicate they are more likely to be ITS), and weighted the data to balance the representation of Caucasian and AA smokers.

### Procedures

All study procedures were reviewed and approved by the University of Pittsburgh Institutional Review Board and written informed consent was obtained from all participants at the start of the study, before beginning any research procedures. Data were collected between November 2007 and April 2010 in Pittsburgh, PA. Subjects engaged in EMA monitoring after participating in 5–6 cue reactivity sessions (see [52]), except for 4 subjects who completed EMA monitoring first. Subjects were provided with a palmtop-computer-based Electronic Diary (ED; Palm Tungsten E2), running specialized software designed for the study (invivodata; Pittsburgh, PA). Subjects received hands-on individual training on the use of the computer, the EMA protocol, and all of the assessments prior to initiating monitoring. Their compliance was monitored, and subjects received feedback during weekly sessions thereafter. Subjects were to engage in EMA for 21 days, but there was some variation in the duration of monitoring, which averaged 21.60 (*SD* = 4.11) days.

The EMA protocols and assessments were identical for DS and ITS, with the exception of algorithms for selecting cigarettes for assessment, as described below. Subjects engaged in event-oriented [46] monitoring of smoking, being instructed to record each cigarette as they initiated it. To avoid excessive subject burden, all cigarettes were recorded, but only a randomly-selected subset was assessed. For DS, for whom assessing all cigarettes was considered unrealistic, the aim was to assess approximately 4 to 5 cigarettes a day, regardless of how many the individual smoked. Accordingly, the ED randomly selected a proportion of reported cigarettes for assessment. The proportion selected for assessment was based on subjects' smoking rates, initially estimated by global self-report and subsequently estimated from the prior day's EMA cigarette entries. (Thus, the sampling proportion could change day-to-day if subjects' smoking fluctuated over days.) For ITS, it was expected that smoking on most days would fall within the 4 to 5 cigarettes targeted for assessment, but with some days exceeding this number. Based on pilot work [55], we expected that ITS might sometimes engage in bouts of smoking in which multiple cigarettes were smoked in a short time, making assessment of all cigarettes unrealistically burdensome. Therefore, the daily algorithm for assessing ITS' cigarettes called for all cigarettes to be assessed until 5 assessments were reached, after which the assessment probability fell to 50%, then fell further to 25% when a cigarette was smoked within 60 minutes of the prior cigarette, indicating a bout of smoking. For both groups, cigarette assessments were weighted by the inverse probability of assessment (recorded by ED), balancing any distortion caused by the sampling scheme (e.g., tendency for cigarettes smoked earlier in the day to have a higher likelihood of assessment among ITS).

Besides recording smoking in real time, participants had two opportunities (at waking and bedtime) to report any cigarettes that they smoked but had not reported in real time. The circumstances of these smoking occasions were not assessed and their timing is not known. Almost all (91%) participants averaged less than 2 “confessional” cigarettes per day during monitoring. These cigarettes were counted in daily totals when analyzing smoking by day of week.

To supplement event-based recording of smoking occasions, ED used signal-based sampling to collect data on non-smoking occasions, by “beeping” subjects at random approximately 4 times per day, with the provision that no non-smoking assessments could fall within 15 minutes of a reported smoking occasion. Empirically, ED issued 3–4 prompts per day on average (DS: *M* = 3.52; ITS: *M* = 3.93). This protocol was active during the entire waking day (though subjects were given an option to “suspend” assessments when necessary – e.g., while driving, in an important meeting). Subjects were to “put ED to sleep” at bedtime, which suppressed beeping, and to “wake up ED” upon arising, at which time random beeping was re-initiated.

### Assessment

All assessments were administered on the computer's touch-screen, with structured responses (no open-ended text) consisting of one of several types: (a) quantitative responses (mood) on a 0–100 point Visual Analog Scale (VAS), where subjects moved a pointer along a line to indicate their response; (b) qualitative responses that required selecting a single alternative (e.g., yes/no, or current location); or (c) qualitative responses that allowed selecting multiple responses (e.g., one could report consuming any combination of food, coffee, alcohol, etc.). ED implemented skip patterns that allowed more detailed inquiry of endorsed responses; e.g., subjects who said they were working were asked to further characterize the nature of the work. Subjects could go back to prior items to edit responses. However, once the assessment ended, responses were stored and no longer accessible for review or change. Assessment data were day- and time-stamped by ED, and uploaded to a server at subject visits.

The assessments covered multiple situational domains. Briefly, in both cigarette and non-smoking assessments, participants were asked questions pertaining to the following: current location, activity, recent food or beverage consumption (including alcohol and caffeinated drinks), whether they were alone or with others, whether others were smoking (and whether those were part of the group of people they were with or just someone in view), and smoking restrictions. On smoking occasions, subjects were also asked whether they had had to move in order to smoke (as when going outside because of smoking restrictions). If they had, the questions focused on the setting that had triggered the decision to smoke. Subjects also rated 14 mood adjectives (able to focus; active; angry/frustrated; bored; calm/relaxed; difficulty concentrating; enthusiastic; happy; irritable; miserable; nervous/tense; quiet/sleepy; restless; sad) on a 0–100 scale, as well as items characterizing overall mood and arousal level. Using factor analyses, the mood data were summarized into several scales: Negative Affect (NA), Positive Affect (PA), Arousal (AR), and Inattention (IA). Factor scores thus derived are standardized scores, and were scaled as T-scores: *M* = 50, *SD* = 10.

### Dataset construction

ED data were examined in conjunction with participant reports to identify periods in which data were clearly invalid, due to problems such as software failure, battery exhaustion, or life circumstances that precluded participation (e.g., when incarcerated). A total of 113 such segments (i.e., spans of corrupt data in the ED data-stream with discernible start and end points, which varied in time length from less than 30 minutes to several days) were deleted, although this retained 98.64% of ED records. In addition, 8 individuals were removed from analysis due to failure to comply with ED protocol (5 completed <50% of prompts, and 3 did not adhere to cigarette entry protocol), as were 8 individuals who provided less than 5 days of data, and one ITS who recorded no smoking during the entire monitoring period and thus contributed no information on smoking patterns. The final dataset comprised 406 participants (212 ITS; 194 DS). DS data consisted of 13,761 smoking and 11,640 non-smoking assessments, and ITS data consisted of 7,778 smoking and 15,290 non-smoking assessments, with participants completing 88% of prompts within the 2 minutes allowed (DS: 87.6%; ITS: 88.2%).

**Figure 1:**
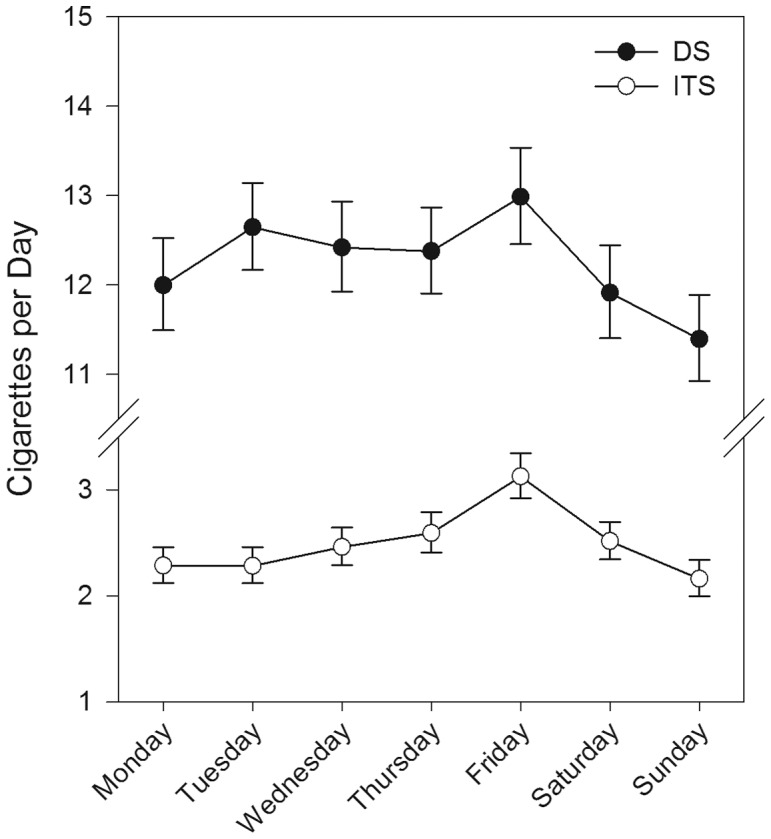
Cigarette consumption by day of week for daily smokers (DS) and intermittent smokers (ITS). Error bars are standard errors.

For analyses of cigarettes per day, all cigarettes reported (including those not recorded in real time) were included, while all partial days (e.g., the first and last days of data collection) were excluded. For time of day analyses, time was divided into 7 blocks (early morning, mid-day, afternoon, evening, night, and late night; see Figure 1), and we computed the smoking rate (cigarettes per hour) for each time block during each participant's waking day.

### Analysis

Observations were weighted to balance two design features that would otherwise distort. Because cigarettes were selected for assessment, and the selection algorithm for ITS favored assessing cigarettes early in the day, smoking observations were weighted by the inverse of their assessment probability, thus equalizing representation of cigarettes. (In other words, those that were under-sampled were up-weighted to bring them up to representative representation.) To balance the oversampling of AA smokers, we also weighted by race to achieve the population proportions of Caucasian and AA ITS and DS (12 subjects of other ethnicity were weighted like Caucasians). Analyses did not adjust for covariates unless otherwise stated.

To assess the relationship between situational factors and smoking, Generalized Estimating Equations (GEE) [56] were used to “predict” smoking (vs. non-smoking) from situational variables. The GEE method accounted for the fact that each subject contributed multiple observations, and that different subjects contributed different numbers of observations. We used the logit link and a first-order auto-regressive correlation structure. For continuous variables, we examined quadratic as well as linear effects. Where appropriate, we assessed models controlling for other stimuli that might be correlated or confounded with the variable of interest; for example, when assessing the effect of being in a bar, we adjusted for drinking alcohol, presence of other smokers, and smoking restrictions. In each case, we assessed the relationship of situational stimuli with smoking within DS and ITS, respectively, and then assessed the situation main effect in both groups combined, as well as the group x stimulus interaction to ascertain differences between DS and ITS in stimulus effects. For time of day and day of week analyses, we also attempted to assess the day x time (and x group) interaction, but those models failed to converge.

Although we did not strictly control for multiplicity in the large number of comparisons, we generally interpreted results as significant only at *p*<.005 or less.

## Results

### Sample characteristics

Table 1 shows the demographic and smoking characteristics of the samples. This is an adult ITS sample, averaging 37 years in age, that has been smoking for an average of 20 years. The ITS have smoked over 40,000 cigarettes on average [15].

**Table 1:** Subject demographics and smoking characteristics.

	Daily Smokers	Intermittent Smokers
	M (SD) or %	M (SD) or %
**Demographics**		
Age	41.18 (11.18)	36.66 (12.44)
Gender (% male)	55.15	49.06
Education (% with post-high school education)	58.25	80.19
*Race* (%)		
African-American	37.63	31.60
Caucasian	59.28	65.57
Other	3.09	2.83
**Smoking characteristics**		
Cigarettes per day (on smoking days)^a^	15.01 (5.86)	4.45 (2.92)
Smoking days per week^a^	—	4.51 (1.64)
FTND^b^ score	5.14 (1.83)	1.42 (1.65)
Years smoked	25.69 (11.83)	19.25 (12.71)
Lifetime cigarettes (1,000 s)	144.87 (98.58)	44.93 (69.79)

*Notes*. M = mean, SD = standard deviation.

a Measured via Time-line Follow-Back interview (Sobell, Sobell, & Maisto, 1979).

b Fagerstrom Test for Nicotine Dependence (Heatherton, Kozlowski, Frecker, & Fagerstrom., 1991).

### Time of day and day of week

**Figure 2:**
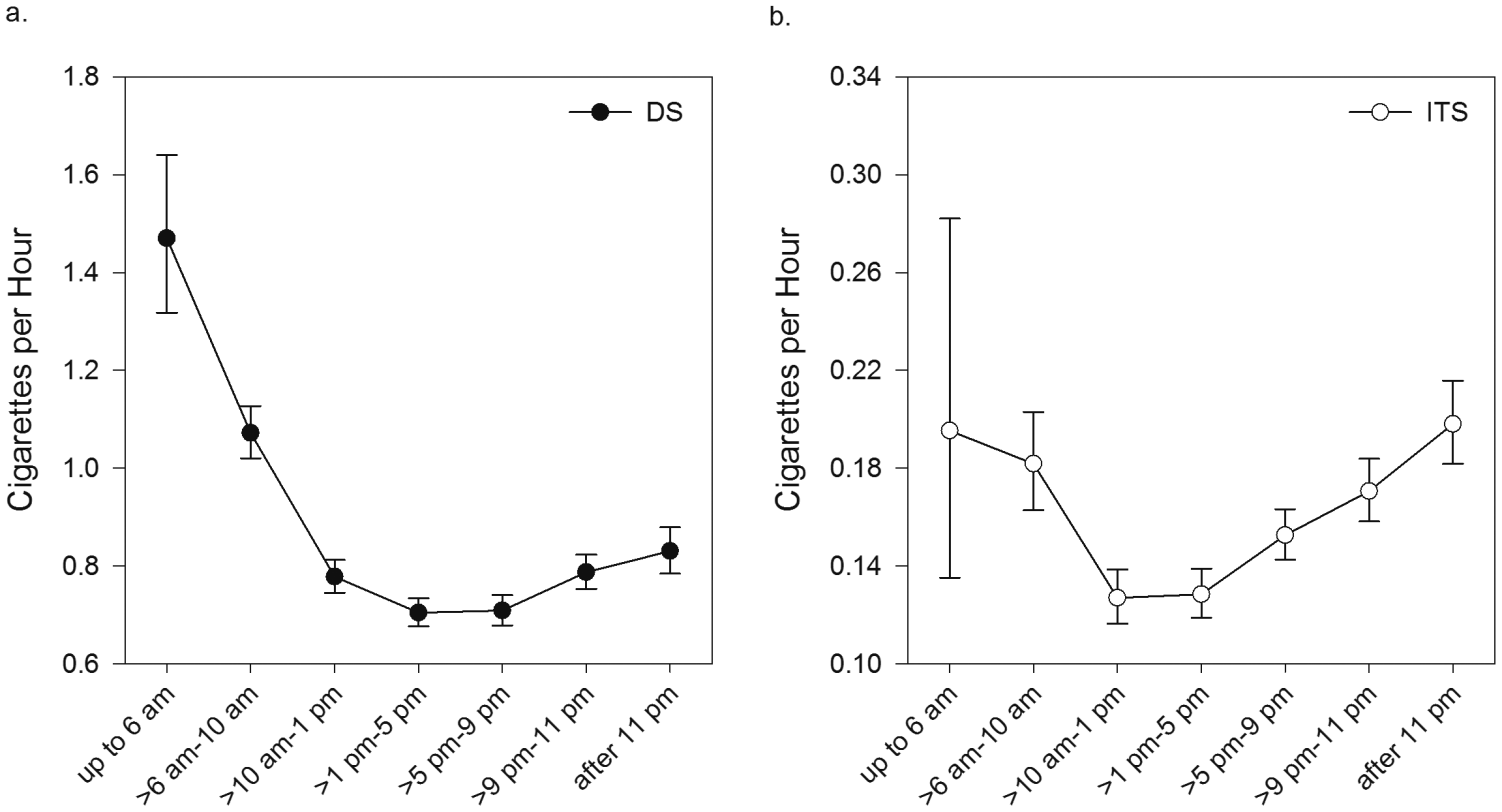
Cigarette consumption by time of day for (a) daily smokers (DS) and (b) intermittent smokers (ITS). Cigarettes consumed within each time block were averaged across all days of the week. Error bars are standard errors. Both means and standard errors are estimated using GEE analysis. Note that the span of y-axes differ between the two panels, with that for DS five times greater than that for ITS; the different axes are necessary to better illustrate the magnitude of the changes within each group.

Smoking rate varied by day of week, differentially for ITS and DS. As shown in Figure 1, both groups increased their cigarette consumption by about 1 cigarette on Fridays (relative to Mondays, the reference day), but this is a significantly greater relative increase for the ITS (interaction *p*<.003). As shown in Figure 2, the smoking rate varied by time of day, with ITS showing a different pattern than DS. DS' smoking rate peaked in the earliest hours of the morning, stayed elevated throughout the morning (relative to 1–5 pm, the reference block), stabilized during the afternoon and evening (1–9 pm), and then rose slightly again at night. In contrast, ITS' rates were stable through most of the morning (at rates significantly higher than during the reference block) and again in the afternoon, but then rose during the evening and night. Both ITS' and DS' smoking was higher in the morning than the afternoon (1–5 pm reference time block), and the groups did not differ in this respect; they differed primarily in that, relative to 1–5 pm, ITS' smoking rose more steeply in the evening (*p*<.001) and late at night (*p*<.006).

#### Location

As seen in Table 2, the association of smoking with particular locations differed by group. In absolute numbers, both DS and ITS smoked more cigarettes at home than at any other location. But accounting for time spent at home, indexed by the non-smoking observations, DS were significantly more likely than ITS to smoke at home. When controlling for the presence of smoking restrictions however, DS were no longer significantly more likely to smoke at home (OR = 1.05 [0.92–1.21], *n.s.*), and ITS likewise became less likely to do so (OR = 0.73 [0.62–0.87], *p*<.0001). In both unadjusted and adjusted analyses, the home x smoker group interactions were significant (*p*<.0007 and *p*<.009, respectively); that is, relative to time spent at home, DS were more likely than ITS to smoke at home.

**Table 2:** Smoker group differences in smoking and nonsmoking occasions by location.

	Daily Smokers				Intermittent Smokers				Situation main effect		Situation x group	
Location	NS (%)	Cig (%)	OR^a^	95% CI	NS (%)	Cig (%)	OR^a^	95% CI	OR	95% CI	OR	95% CI
**Home**	**56.0**	**57.6**	**1.33** ***	**[1.15–1.53]**	**53.1**	**41.0**	**0.87**	**[0.74–1.04]**	**1.24** **	**[1.08–1.42]**	**0.67** **	**[0.53–0.84]**
**Bar**	**1.5**	**3.0**	**2.10** ***	**[1.54–2.87]**	**1.7**	**13.8**	**6.24** ***	**[4.50–8.65]**	**2.85** ***	**[2.16–3.75]**	**2.93** ***	**[1.83–4.68]**
**Restaurant**	**1.3**	**1.3**	**0.85**	**[0.60–1.20]**	**1.9**	**1.7**	**0.82**	**[0.64–1.05]**	**0.80**	**[0.62–1.04]**	**0.92**	**[0.59–1.43]**
**Others' home**	**7.3**	**6.7**	**0.86**	**[0.71–1.04]**	**8.9**	**11.3**	**1.09**	**[0.89–1.33]**	**0.83** *	**[0.68–1.00]**	**1.28**	**[0.97–1.70]**
**Workplace**	**16.0**	**10.1**	**0.47** ***	**[0.39–0.58]**	**17.1**	**9.4**	**0.44** ***	**[0.35–0.55]**	**0.47** ***	**[0.39–0.55]**	**0.93**	**[0.68–1.28]**
**Outside**	**10.6**	**16.9**	**1.57** ***	**[1.32–1.88]**	**8.5**	**16.4**	**2.01** ***	**[1.71–2.35]**	**1.86** ***	**[1.60–2.17]**	**1.25**	**[0.98–1.60]**
**Vehicle**	**3.1**	**1.9**	**0.46** ***	**[0.34–0.63]**	**4.6**	**3.4**	**0.71** *	**[0.52–0.97]**	**0.48** ***	**[0.39–0.60]**	**1.50**	**[0.95–2.37]**
**Other**	**4.2**	**2.6**	**0.48** ***	**[0.38–0.61]**	**4.3**	**3.1**	**0.56** ***	**[0.45–0.71]**	**0.50** ***	**[0.39–0.63]**	**1.17**	**[0.83–1.64]**

*Notes*.

*p<.05.

**p<.005.

***p<.0005.

NS = Not smoking, Cig = Smoking, OR = odds ratio, CI = confidence interval.

Percentages were derived by averaging across subjects the within-subject computed means. All analyses were weighted by race. Smoking observations were also weighted by inverse probability of assessment. ORs are calculated by GEE.

a ORs and descriptive statistics may not be consistent with each other, due to internal weighting inherent in GEE analysis.

Both groups were more likely to smoke when in a bar, but the effect was three times stronger among ITS, and the differences remained after controlling for alcohol consumption, smoking restrictions, and others' smoking (interaction *p*<.0001). Smoking was associated with other locations, but not differentially for ITS vs DS. Both groups were less likely to smoke in the workplace, even after accounting for smoking restrictions and others' smoking (DS: OR = 0.63 [0.51–0.78], *p*<.0001; ITS: OR = 0.58 [0.46–0.73], *p*<.0001). Both groups were also more likely to smoke when outdoors, but less likely to smoke in “other” locations (not otherwise classified).

#### Activities

As shown in Table 3, participants in both groups were less likely to smoke when they were working; this was particularly so for ITS, once we controlled for smoking restrictions (OR = 0.55 [0.47–0.64], *p*<.0001). All kinds of work (including, e.g., personal chores) suppressed smoking among ITS, but only jobs and school-work did so among DS. Leisure activities did not differentially affect DS and ITS, except for media consumption, which affected the groups differently: DS significantly increased smoking when consuming media, whereas ITS were (nonsignificantly) less likely to smoke when consuming media. Interacting with others, especially socializing, significantly increased the likelihood of smoking among ITS, more so than among DS. Both groups increased their odds of smoking by at least 30% when they were in between activities.

**Table 3:** Smoker group differences in smoking and nonsmoking occasions by activity.

	Daily Smokers				Intermittent Smokers				Situation main effect		Situation x group	
Activity	NS (%)	Cig (%)	OR^a^	95% CI	NS (%)	Cig (%)	OR^a^	95% CI	OR	95% CI	OR	95% CI
Working	**24.6**	**17.6**	**0.59** ***	**[0.51–0.69]**	**28.7**	**15.3**	**0.48** ***	**[0.41–0.56]**	**0.55** ***	**[0.48–0.62]**	**0.80**	**[0.64–1.00]**
***Type of work*** b **(ref = not working)**												
**Job**	**15.8**	**10.0**	**0.47** ***	**[0.38–0.59]**	**17.0**	**7.5**	**0.37** ***	**[0.29–0.47]**	**0.45** ***	**[0.37–0.54]**	**0.77**	**[0.55–1.08]**
**School**	**1.5**	**1.0**	**0.39** ***	**[0.28–0.55]**	**2.9**	**1.7**	**0.36** ***	**[0.25–0.51]**	**0.34** ***	**[0.25–0.47]**	**0.88**	**[0.52–1.47]**
**House/personal**	**6.5**	**6.0**	**0.94**	**[0.78–1.15]**	**8.3**	**5.8**	**0.73** ***	**[0.61–0.87]**	**0.80** *	**[0.68–0.95]**	**0.78**	**[0.60–1.02]**
**Other**	**0.8**	**0.6**	**0.65** *	**[0.44–0.97]**	**0.5**	**0.2**	**0.42** ***	**[0.26–0.68]**	**0.69**	**[0.46–1.03]**	**0.64**	**[0.34–1.22]**
Leisure	**38.1**	**39.3**	**1.21** **	**[1.06–1.38]**	**36.1**	**34.3**	**0.99**	**[0.89–1.11]**	**1.15** *	**[1.02–1.29]**	**0.82** *	**[0.69–0.99]**
***Type of leisure*** b **(ref = not engaged in leisure)**												
**Hobbies**	**1.4**	**1.1**	**0.88**	**[0.61–1.26]**	**2.6**	**1.6**	**0.76**	**[0.52–1.11]**	**0.72** *	**[0.53–1.00]**	**0.82**	**[0.48–1.42]**
**Media**	**16.5**	**16.6**	**1.31** *	**[1.07–1.62]**	**14.0**	**9.4**	**0.82**	**[0.66–1.02]**	**1.24** *	**[1.02–1.51]**	**0.62** **	**[0.45–0.85]**
**Sports/exercise**	**0.4**	**0.4**	**1.41**	**[0.77–2.57]**	**0.7**	**0.5**	**1.29**	**[0.58–2.85]**	**1.11**	**[0.71–1.74]**	**0.97**	**[0.34–2.81]**
**Hanging out**	**7.5**	**9.4**	**1.22** *	**[1.05–1.43]**	**9.1**	**14.5**	**1.27** *	**[1.06–1.54]**	**1.13**	**[0.97–1.32]**	**1.04**	**[0.81–1.35]**
**Waiting**	**1.9**	**2.2**	**1.41**	**[1.06–1.88]**	**1.4**	**1.5**	**1.22**	**[0.95–1.56]**	**1.39** *	**[1.10–1.74]**	**0.87**	**[0.59–1.29]**
**Doing nothing**	**8.0**	**7.6**	**1.08**	**[0.91–1.29]**	**5.8**	**5.2**	**1.09**	**[0.84–1.42]**	**1.15**	**[0.97–1.35]**	**1.04**	**[0.75–1.45]**
**Other**	**2.5**	**2.0**	**0.94**	**[0.69–1.29]**	**2.6**	**1.5**	**0.78** *	**[0.63–0.97]**	**0.89**	**[0.69–1.15]**	**0.84**	**[0.57–1.24]**
Interacting with others	**11.9**	**12.8**	**1.10**	**[0.94–1.29]**	**16.2**	**25.2**	**1.48** ***	**[1.24–1.77]**	**1.04**	**[0.90–1.19]**	**1.36** *	**[1.06–1.74]**
***Type of interaction*** b **(ref = not interacting with others)**												
**Socializing**	**8.4**	**9.9**	**1.15**	**[0.97–1.37]**	**11.8**	**21.7**	**1.67** ***	**[1.37–2.03]**	**1.11**	**[0.96–1.28]**	**1.46** *	**[1.11–1.91]**
**For work**	**1.0**	**0.6**	**0.88**	**[0.56–1.40]**	**1.5**	**1.0**	**0.74**	**[0.46–1.20]**	**0.67**	**[0.42–1.08]**	**0.81**	**[0.41–1.60]**
**Household**	**1.0**	**1.1**	**1.29**	**[0.97–1.71]**	**0.9**	**0.9**	**1.25**	**[0.86–1.79]**	**1.26**	**[0.95–1.66]**	**0.99**	**[0.62–1.58]**
**Arguing**	**0.2**	**0.3**	**1.13**	**[0.60–2.10]**	**0.3**	**0.5**	**1.56**	**[0.71–3.45]**	**1.05**	**[0.66–1.67]**	**1.38**	**[0.49–3.92]**
**Other**	**1.3**	**1.0**	**0.78**	**[0.53–1.14]**	**1.7**	**1.1**	**0.99**	**[0.73–1.36]**	**0.73**	**[0.55–0.98]**	**1.31**	**[0.79–2.18]**
Between activities	**15.3**	**20.3**	**1.41** ***	**[1.24–1.61]**	**15.6**	**19.0**	**1.30** ***	**[1.15–1.48]**	**1.40** ***	**[1.23–1.60]**	**0.91**	**[0.75–1.10]**
Other activities	**15.3**	**14.3**	**0.85**	**[0.70–1.04]**	**11.4**	**9.2**	**0.88**	**[0.73–1.04]**	**0.94**	**[0.78–1.13]**	**1.06**	**[0.81–1.40]**

*Notes*.

*p<.05.

**p<.005.

***p<.0005.

NS = Not smoking, Cig = Smoking, OR = odds ratio, CI = confidence interval, ref = reference group.

Percentages were derived by averaging across subjects the within-subject computed means. All analyses were weighted by race. Smoking observations were also weighted by inverse probability of assessment. ORs are calculated by GEE.

a ORs and descriptive statistics may not be consistent with each other, due to internal weighting inherent in GEE analysis.

b All subcategories within this situational domain were treated as a single model in GEE analysis.

#### Food, caffeine, and alcohol consumption

As displayed in Table 4, consumption of caffeinated drinks was similarly associated with increased smoking in both groups, but the groups reacted very differently when drinking alcohol. Both groups' smoking was substantially increased when drinking alcohol, but the effect was substantially greater among ITS, whose odds of smoking were increased by 300% (vs. a 104% increase among DS). The overall association between smoking and alcohol remained after controlling for a variety of correlated factors (i.e., location, time of day, others smoking, restrictions, and engaged in leisure activity), but such controls nearly eliminated the difference in between groups in the strength of the association t(DS: OR = 1.64 [1.25–2.15], *p*<.0003; ITS: OR = 1.77 [1.36–2.29], *p*<.0001). In other words, once these contextual factors were accounted for, drinking alcohol did not seem to have a disproportionate influence on ITS smoking compared to DS'. Despite the strong effect of alcohol on ITS' relative probability of smoking, only 20% of ITS' total cigarettes were smoked within 15 minutes of drinking.

**Table 4:** Smoker group differences in smoking and nonsmoking occasions by consumption of food and drink.

	Daily Smokers				Intermittent Smokers				Situation main effect		Situation x group	
Consumption	NS (%)	Cig (%)	OR^a^	95% CI	NS (%)	Cig (%)	OR^a^	95% CI	OR	95% CI	OR	95% CI
Eating and/or drinking	**10.7**	**14.7**	**1.61** ***	**[1.36–1.91]**	**13.2**	**28.2**	**2.22** ***	**[1.87–2.64]**	**1.55** ***	**[1.34–1.79]**	**1.40** *	**[1.09–1.80]**
***Type of food or drink consumption***												
**Food**	**6.3**	**6.8**	**1.23** *	**[1.02–1.47]**	**8.3**	**9.8**	**1.30** *	**[1.04–1.64]**	**1.11**	**[0.96–1.29]**	**1.07**	**[0.79–1.46]**
**Caffeinated drink**	**4.1**	**6.3**	**1.79** ***	**[1.40–2.28]**	**4.1**	**5.2**	**1.75** ***	**[1.34–2.30]**	**1.78** ***	**[1.44–2.20]**	**0.98**	**[0.67–1.44]**
**Non-caffeinated drink**	**2.9**	**2.8**	**1.14**	**[0.81–1.61]**	**3.7**	**2.9**	**1.02**	**[0.78–1.34]**	**0.99**	**[0.73–1.34]**	**0.87**	**[0.55–1.37]**
**Alcohol**	**2.7**	**4.9**	**2.04** ***	**[1.61–2.57]**	**3.9**	**20.1**	**4.00** ***	**[3.06–5.24]**	**2.12** ***	**[1.69–2.65]**	**1.96** ***	**[1.36–2.84]**

*Notes*.

*p<.05.

**p<.005.

***p<.0005.

NS = Not smoking, Cig = Smoking, OR = odds ratio, CI = confidence interval.

Percentages were derived by averaging across subjects the within-subject computed means. All analyses were weighted by race. Smoking observations were also weighted by inverse probability of assessment. ORs are calculated by GEE.

a ORs and descriptive statistics may not be consistent with each other, due to internal weighting inherent in GEE analysis.

#### Social setting

As detailed in Table 5, ITS smoked 37% of their cigarettes when alone (compared to 52% among DS). Both groups were similar in being significantly *more* likely to smoke when alone than when with others, once the influence of others' smoking was controlled (DS: OR = 2.02 [1.77–2.31], *p*<.0001; ITS: OR = 2.12 [1.70–2.63], *p*<.0001). ITS were also more likely to smoke when friends (60% increase) or acquaintances (75% increase) were present; this was not true of DS. For both groups, being with a co-worker halved the odds of smoking, while spouses had no apparent effect on smoking.

**Table 5:** Smoker group differences in smoking and nonsmoking occasions by social setting.

	Daily Smokers				Intermittent Smokers				Situation main effect		Situation x group	
Social setting	NS (%)	Cig (%)	OR^a^	95% CI	NS (%)	Cig (%)	OR^a^	95% CI	OR	95% CI	OR	95% CI
***Presence of others***												
**Alone**	**46.4**	**52.2**	**1.45** ***	**[1.29–1.63]**	**41.6**	**37.0**	**1.12**	**[0.92–1.37]**	**1.47** ***	**[1.30–1.66]**	**0.79** *	**[0.60–0.98]**
**With friends**	**15.0**	**16.2**	**1.03**	**[0.86–1.23]**	**18.4**	**36.5**	**1.60** ***	**[1.33–1.93]**	**1.05**	**[0.90–1.22]**	**1.55** **	**[1.19–2.03]**
**With acquaintances**	**6.5**	**5.9**	**0.90**	**[0.73–1.12]**	**6.5**	**12.0**	**1.75** ***	**[1.38–2.23]**	**1.04**	**[0.88–1.23]**	**2.00** ***	**[1.44–2.79]**
**With family**	**17.5**	**14.6**	**0.81** *	**[0.69–0.95]**	**16.3**	**12.0**	**0.92**	**[0.73–1.17]**	**0.86**	**[0.73–1.01]**	**1.17**	**[0.88–1.57]**
**With coworkers**	**11.6**	**6.6**	**0.47** ***	**[0.37–0.59]**	**12.6**	**7.5**	**0.47** ***	**[0.37–0.60]**	**0.47** ***	**[0.38–0.57]**	**1.01**	**[0.72–1.42]**
**With spouse**	**15.2**	**15.8**	**0.91**	**[0.76–1.10]**	**17.2**	**16.2**	**0.85**	**[0.63–1.14]**	**0.84**	**[0.69–1.03]**	**0.93**	**[0.65–1.33]**
***Presence of other smokers***												
Others smoking	**17.0**	**28.0**	**2.08** ***	**[1.69–2.50]**	**10.1**	**42.9**	**5.26** ***	**[4.17–6.67]**	**3.13** ***	**[2.63–3.70]**	**2.44** ***	**[1.82–3.23]**
**Other smokers in group**	**10.6**	**17.0**	**1.71** ***	**[1.37–2.14]**	**6.7**	**31.9**	**5.44** ***	**[4.30–6.90]**	**2.74** ***	**[2.28–3.29]**	**3.02** ***	**[2.16–4.22]**
**Other smokers in view**	**8.9**	**15.1**	**2.23** ***	**[1.88–2.66]**	**4.8**	**20.0**	**4.56** ***	**[3.71–5.61]**	**3.12** ***	**[2.67–3.65]**	**2.02** ***	**[1.53–2.67]**
***Specific comparisons between exclusive subcategories in which other smokers were present*** b **(ref = no other smokers present)**												
**In group only**	**8.1**	**13.0**	**1.74** ***	**[1.35–2.24]**	**5.3**	**22.9**	**4.95** ***	**[3.76–6.52]**	**2.71** ***	**[2.20–3.33]**	**2.68** ***	**[1.83–3.93]**
**In view only**	**6.5**	**11.0**	**2.41** ***	**[1.99–2.92]**	**3.4**	**11.0**	**3.84** ***	**[3.09–4.77]**	**3.25** ***	**[2.71–3.90]**	**1.57** **	**[1.17–2.11]**
**Both in group and in view**	**2.5**	**4.1**	**2.31** ***	**[1.77–3.02]**	**1.4**	**9.0**	**10.19** ***	**[7.31–14.21]**	**4.22** ***	**[3.35–5.32]**	**4.14** ***	**[2.67–6.41]**

*Notes*.

*p<.05.

**p<.005.

***p<.0005.

NS = Not smoking, Cig = Smoking, OR = odds ratio, CI = confidence interval, ref = reference group.

Percentages were derived by averaging across subjects the within-subject computed means. All analyses were weighted by race. Smoking observations were also weighted by inverse probability of assessment. ORs are calculated by GEE.

a ORs and descriptive statistics may not be consistent with each other, due to internal weighting inherent in GEE analysis.

b All subcategories within this situational domain were treated as a single model in GEE analysis.

The presence of others smoking increased smoking in both groups, but the effects were 2 to 3 times larger for ITS than for DS. This effect was evident whether the people smoking were people in view, or were part of one's group (e.g., people one is out to dinner with). However, ITS appear to be most influenced by smokers in their social group, whereas DS appear to be most influenced by seeing strangers smoking.

#### Cigarette availability

As seen in Table 6, ITS were particularly likely to smoke when cigarettes were easily available; these situations were associated with a 28-fold increase in their odds of smoking (vs. 11-fold for DS). DS were much more likely than ITS to report that cigarettes were easily available, even when they were not smoking.

**Table 6:** Smoker group differences in smoking and nonsmoking occasions by smoking contexts.

	Daily Smokers				Intermittent Smokers				Situation main effect		Situation x group	
Smoking context	NS (%)	Cig (%)	OR^a^	95% CI	NS (%)	Cig (%)	OR^a^	95% CI	OR	95% CI	OR	95% CI
***Cigarette availability*** b **(ref = cigarettes not available)**												
**Easily available**	**91.1**	**97.0**	**11.00** ***	**[7.08–17.08]**	**65.6**	**91.9**	**28.31** ***	**[17.6–45.54]**	**27.68** ***	**[20.17–38.0]**	**2.59** **	**[1.36–4.95]**
**Available with difficulty**	**4.1**	**2.2**	**5.42** ***	**[3.23–9.09]**	**11.4**	**5.9**	**6.96** ***	**[4.06–11.94]**	**6.84** ***	**[4.61–10.16]**	**1.30**	**[0.61–2.75]**
***Smoking restrictions*** b **(ref = smoking allowed)**												
**Discouraged**	**7.9**	**6.6**	**0.65** *	**[0.43–0.98]**	**19.5**	**11.4**	**0.43** ***	**[0.31–0.59]**	**0.37** ***	**[0.26–0.52]**	**0.67**	**[0.40–1.14]**
**Forbidden**	**19.5**	**11.4**	**0.37** ***	**[0.30–0.46]**	**21.2**	**10.5**	**0.29** ***	**[0.23–0.37]**	**0.32** ***	**[0.26–0.39]**	**0.82**	**[0.59–1.13]**
***Reason smoking forbidden*** b **(ref = not forbidden)**												
**By law**	**12.7**	**7.0**	**0.36** ***	**[0.28–0.46]**	**11.9**	**7.1**	**0.32** ***	**[0.25–0.40]**	**0.33** ***	**[0.26–0.41]**	**0.91**	**[0.64–1.30]**
**Own rule**	**5.4**	**4.3**	**0.46** ***	**[0.32–0.68]**	**13.3**	**7.1**	**0.40** ***	**[0.26–0.62]**	**0.29** ***	**[0.21–0.41]**	**0.91**	**[0.51–1.64]**
**Other's rule**	**9.3**	**6.7**	**0.57** **	**[0.39–0.83]**	**15.5**	**7.7**	**0.34** ***	**[0.25–0.46]**	**0.38** ***	**[0.27–0.53]**	**0.61**	**[0.38–1.00]**

*Notes*.

*p<.05.

**p<.005.

***p<.0005.

NS = Not smoking, Cig = Smoking, OR = odds ratio, CI = confidence interval, ref = reference group.

Percentages were derived by averaging across subjects the within-subject computed means. All analyses were weighted by race. Smoking observations were also weighted by inverse probability of assessment. ORs are calculated by GEE.

a ORs and descriptive statistics may not be consistent with each other, due to internal weighting inherent in GEE analysis.

b All subcategories within this situational domain were treated as a single model in GEE analysis.

#### Smoking restrictions

ITS' and DS' smoking were equally affected when smoking was forbidden, which reduced the odds of smoking by more than 60% (Table 6). This was largely unchanged by controlling for others smoking (DS: OR = 0.39 [0.31–0.48], *p*<.0001; ITS: OR = 0.38 [0.29–0.48], *p*<.0001). Notably, ITS were more likely than DS to cite self-imposed restrictions and restrictions imposed by others' preferences when they were not smoking.

#### Mood

**Figure 3:**
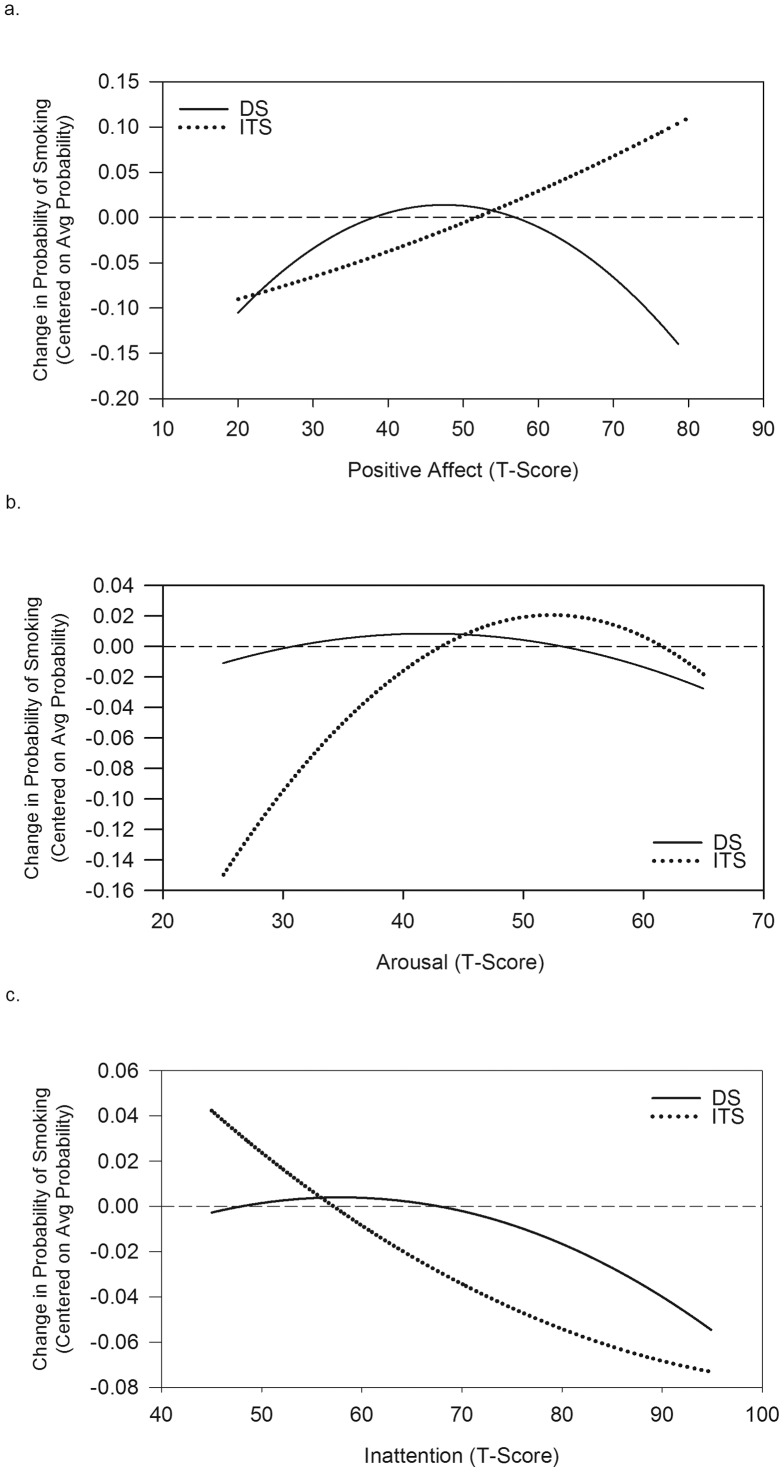
Modeled associations between mood measures and changes in the probability of smoking (vs. randomly-selected non-smoking occasions). Data are presented as changes relative to the average probability of smoking, for daily smokers (DS) and intermittent smokers (ITS) separately, because the absolute probability is influenced by the sampling scheme for smoking and non-smoking occasions, which differs between groups. The mood scales presented are (a) Positive Affect; (b) Arousal; (c) Inattention. All mood scores are standardized factor scores scaled to *M* = 50, *SD* = 10. In each case, the range of the mood score represents the range encompassing approximately 95% of the observed scores.

Relationships between mood and smoking were complex. As seen in Table 7, only one interaction reached the designated level of significance. As Figure 3 demonstrates, among DS, the probability of smoking initially increased with increased PA, then decreased. In contrast, ITS showed a steady increase in smoking with increased PA. NA had no significant association with smoking in either group.

**Table 7:** Smoker group differences in smoking and nonsmoking occasions by mood states.

	Daily Smokers				Intermittent Smokers				Mood main effect		Mood x group	
Mood	NS M ± SD	Cig M ± SD	OR^a^	95% CI	NS M ± SD	Cig M ± SD	OR^a^	95% CI	OR^a^	95% CI	OR^a^	95% CI
**Negative affect**	**51.7±7.7**	**51.1±7.6**	**1.00**	**[0.92–1.09]**	**52.2±7.3**	**52.0±7.5**	**0.91**	**[0.82–1.02]**	**0.95**	**[0.86–1.04]**	**0.92**	**[0.80–1.06]**
***Quadratic***			**1.01**	**[0.97–1.06]**			**1.08** *	**[1.02–1.14]**	**1.06** *	**[1.01–1.11]**	**0.99**	**[0.98–1.01]**
**Positive affect**	**49.9±6.1**	**50.1±6.0**	**0.98**	**[0.90–1.07]**	**50.7±6.3**	**51.6±6.2**	**1.16** *	**[1.04–1.29]**	**0.96**	**[0.88–1.04]**	**1.19** *	**[1.03–1.37]**
***Quadratic***			**0.92** **	**[0.87–0.96]**			**1.00**	**[0.94–1.07]**	**0.93** **	**[0.89–0.98]**	**1.02** **	**[1.01–1.04]**
**Arousal**	**48.7±5.3**	**48.8±5.0**	**0.97**	**[0.90–1.04]**	**48.7±4.5**	**50.3±5.3**	**1.11** *	**[1.03–1.19]**	**1.00**	**[0.93–1.07]**	**1.14** *	**[1.03–1.27]**
***Quadratic***			**0.96**	**[0.92–1.01]**			**0.90** ***	**[0.86–0.94]**	**0.96**	**[0.91–1.01]**	**1.01** *	**[1.00–1.03]**
**Inattention**	**51.8±8.6**	**51.4±8.4**	**0.99**	**[0.93–1.07]**	**53.3±9.4**	**52.8±9.4**	**0.88** **	**[0.81–0.94]**	**0.94**	**[0.87–1.00]**	**0.88** *	**[0.79–0.98]**
***Quadratic***			**0.98**	**[0.94–1.01]**			**1.01**	**[0.98–1.05]**	**0.98**	**[0.95–1.01]**	**0.99** *	**[0.98–1.00]**

*Notes*.

*p<.05.

**p<.005.

***p<.0005.

NS = Not smoking, Cig = Smoking, OR = odds ratio, CI = confidence interval.

Means were derived by averaging across subjects the within-subject computed means. All analyses were weighted by race. Smoking observations were also weighted by inverse probability of assessment. ORs are calculated by GEE. For each variable, the first row lists the linear effects; quadratic effects for each are listed in a second row.

a Mood variables were captured on a 0–100 scale. Values were divided by 10 in order to obtain ORs that reflect a 10-point change in mood (rather than a 1-point change).

Although no other interactions met the criteria for significance, there were several significant effects evident among ITS but not DS. ITS' smoking probability declined with increased IA, and increased to a point with increasing AR, then flattened out. (The AR effect remained after controlling for other influences on AR, such as time of day, activity, location, and social setting.) These IA and AR effects seemed largely independent of each other, and of time of day. Controlling for other moods or for time of day in multivariate models (not shown) generally did not materially change the mood results.

## Discussion

Detailed data on the real-world contexts of smoking, collected by real-time EMA methods, demonstrated both substantial differences and important similarities in smoking patterns of DS and ITS. Compared to DS, ITS' smoking was more strongly associated with being away from home, in bars, drinking alcohol, engaged in social interaction, with friends and acquaintances, and where others were smoking. This pattern suggests a profile of “indulgent smoking” [12] in situations where smoking might enhance an already pleasant setting.

A number of situational variables affected DS and ITS similarly. Neither group's smoking was reliably influenced by negative affect, or by drinking non-caffeinated drinks, eating food, or being in others' homes. On the other hand, both were more likely to smoke when alone, once the influence of others' smoking was removed. Both were less likely to smoke when at the workplace, with coworkers, and/or engaged in a job or schoolwork. Both were more likely to smoke when drinking caffeinated drinks or when in between activities. Both were far less likely to smoke when smoking was forbidden. Even where these similarities were seen, however, ITS showed stronger links between context and smoking. For example, ITS showed stronger links between smoking and situational variables such as being at a bar, drinking alcohol, and being where others are smoking. Overall, ITS' and DS' smoking seemed to respond to similar environmental cues, but the associations were consistently stronger among ITS.

Turning to the particular variables that more strongly influenced ITS' smoking: as noted above, smoking when with friends or acquaintances when feeling good, at a bar, drinking alcohol, etc., is consistent with the image of ITS as “indulgent” smokers [12], [51] who smoke when smoking is pleasurable, to enhance an already-rewarding experience. This pattern resembles that described in tobacco industry documents for “social smokers” [8], [27], loosely defined. Recent work has defined social smokers more carefully and behaviorally [10]. Application of these definitions to ITS smokers ;will require deeper analyses of subgroups, as these analyses focus on trends in the group as a whole, which reflect a mixture of heterogeneous patterns and perhaps subgroups, some of whom may be social smokers. Notably, although the relative frequency of situational contexts that could reflect social smoking is elevated, the absolute frequency of the relevant conditions is low: ITS only smoked 20% of their cigarettes when drinking alcohol; 25% when interacting with others; and less than half when others were smoking.

Even though ITS were more likely to smoke when others were present, they actually smoked more than a third of their cigarettes while alone. This last finding strongly suggests that ITS' smoking motives, as a group, are not wholly social, even if their self-image were that of a social smoker [10]. That they smoke so many of their cigarettes when they are alone suggests that at least some ITS have intrinsic, non-social motives for smoking, likely related to the reinforcing pharmacological effects of nicotine, which are not directly addressed by these analyses. Also inconsistent with the image of ITS as generally being social or party smokers [27] is the fact that their cigarette consumption was as high in the morning as at night (and higher than their afternoon smoking rate). The prevalence of morning smoking among ITS is striking. In DS, morning smoking is typically attributed to dependence and the need to replenish nicotine following its overnight clearance [57]. However, ITS' morning smoking might be driven by cues associated with morning activities or by instrumental needs that arise in the morning (e.g., nicotine-related increases in arousal or cognitive capacity).

We saw relatively few and small effects due to mood. Whereas DS' smoking increased with PA up to a point, then decreased, ITS showed steady increases with increased PA – the reverse of “negative affect smoking,” and perhaps related to nicotine's ability to enhance reinforcing experiences [33]. Contrary to ITS' questionnaire reports that they smoke more when stressed or upset [15], the EMA data showed no relationship of smoking to NA. Among ITS, the probability of smoking dropped with increased IA, making it unlikely that ITS generally smoke to improve cognitive performance (though it does not preclude some doing so in particular contexts). Indeed, the data suggested that smoking might be used to moderate high-arousal states [34], as the probability of smoking was higher when ITS were experiencing high arousal, even after controlling for other influences on arousal.

Importantly, NA was unrelated to smoking among DS, confirming findings from two other EMA studies of DS [24], [58], both of which contradicted the common belief of both smokers and researchers that people smoke when upset [32]. This study adds to those previous in that it involved a large sample of DS who were not trying to quit and included a large range of smoking rates, from 5 cigarettes per day to 30 per day, averaging 15 per day, the national average. As noted in Shiffman et al. [59], this does not mean that *no* smokers smoke when upset – only that this is not a common pattern, which contradicts the usual assertion that “negative-affect smoking” is a major common driver of ad-libitum smoking.

The present findings on ITS parallel earlier findings on a small sample of “chippers” – individuals who smoke at very low rates, even if smoking daily [60], [61]. EMA data also showed that chippers smoke in “indulgent” situations associated with drinking and social activity [51]. This may suggest continuity between low-rate daily smoking and non-daily smoking, and also links the currently prevalent phenomenon of ITS with the then-rare phenomenon of low-rate smoking 25 years ago.

The findings from this study were not consistent with those from laboratory cue reactivity studies of ITS [40], [45], [62]. Whereas the EMA data showed robust increases in the likelihood of smoking when others were smoking and when alcohol was consumed, no effects on smoking were seen after laboratory exposures to smoking or alcohol cues [45]. Moreover, in the laboratory, ITS were no more responsive to cues than were DS [40], [45], [62]. Perhaps the laboratory simulation does not fully represent real-world experiences – actually being around others smoking is different than seeing pictures of others smoking, and actually consuming alcohol is different than seeing pictures of alcoholic drinks. Smoking triggers may also be different in the lab than in life, suggesting the need to assess the relationship between laboratory cue reactivity responses and real-world behavior.

A good deal of research on non-daily smoking has been conducted in populations of adolescents and young adults [9], [62], [63], [64]. That literature has suggested that smoking patterns in those populations are dominated by social and party smoking, and limited to a few settings. In contrast, this study suggested that ITS smoking occurs in a wider variety of settings, including in the morning and when alone. In interpreting the present findings in relation to that literature, it is important to remember that this is an adult sample of ITS whose average age was near 40, and who have, on average, been smoking for decades. Thus, it is not surprising that their smoking patterns would be more diverse and would not correspond closely to those seen at younger ages and earlier stages in smoking careers. As non-daily smoking is becoming more prevalent in the adult smoking population, it will be important to distinguish these “mature” patterns from those seen in adolescents and young adults.

### Limitations

This study suffered from some limitations. Although EMA methods have advantages, they still rely on self-report; subjects could have deliberately or unintentionally misrepresented their state or behavior. Subject classification as DS or ITS was also based on self-report, which could be incorrect. Smoking restrictions also likely distort the associations that might otherwise have held, but the influence of restrictions is a real-world fact shaping current smoking patterns. Non-compliance with the EMA protocol had potential to bias the data; for example, if subjects failed to respond to prompts under particular conditions, such as when they were stressed. The EMA records showed that subjects responded to the vast majority of ED-issued prompts in a timely way. However, we have no reliable way of knowing whether or when subjects may have failed to record episodes of smoking, or to record them in a timely way, which could also skew the results. An analysis of biochemical markers in another study suggested that under-reporting was a minor issue [24], [65], but it cannot be eliminated as a concern. Another concern with intensive EMA monitoring is reactivity – the possibility that the monitoring itself changes behavior. However, McFall [66] found that self-monitoring of smoking affected smoking only when subjects were trying to change their smoking, and these subjects were not. Previous analyses of smoking [24] and other behaviors [67], [68] have also found at most modest reactivity to EMA.

Our analyses are also limited by the content of the EMA assessments. For example, the data showed that ITS were less likely to smoke when consuming media. Yet, studies in adolescents and young adults have shown that exposure to media such as movies that portray smoking, can promote uptake of smoking [69], [70] and directly prompt smoking acutely [70]. However, we did not assess what media were being consumed, or the smoking content of such media, so our data do not address that question. The present analyses also did not address some interesting questions about heterogeneity within ITS; for example, heavier-smoking ITS, or those who previously had been daily smokers (see [15]) may differ in their smoking patterns. There may also be subgroups of interest based on their smoking patterns themselves, such as social smokers, who might be identified on the basis of their EMA data. Finally, we made many statistical comparisons, which may have inflated the Type I error rate. Accordingly, findings, especially those with more modest p-values, must be treated with caution.

At the same time, the study had considerable strengths. The EMA method allowed for very detailed characterization of smoking settings, without relying on subjects' memory or their global impressions of smoking patterns. The availability of data from non-smoking moments allowed for use of a case-cross-over design, with true evaluation of the within-person association between context and smoking [48], controlling for overall subject characteristics (e.g., frequency of drinking, regardless of smoking). The data were collected using items that have been tested in previous studies, and mood was assessed with factor-analysis-based multi-item scales. The sample of smokers included considerable diversity, covering a large range of smoking rates. Subjects were not trying to quit, so the data should represent typical ad libitum smoking.

Some of the findings reported here have policy implications. Notably, environmental smoking restrictions were effective – even if not perfectly so – in suppressing smoking in those prohibited contexts (see also [24], [71]). However, it is not clear how much they suppressed cigarette consumption overall: the cigarettes foregone in restricted settings might have been made up when smoking was allowed, as suggested by some [72], [73], [74]. Restrictions could potentially have a greater effects on ITS, since their smoking is more concentrated in certain locations, such as bars. Restrictions on smoking in bars were not widely in place at the time of this study, but their subsequent imposition could reduce ITS (and DS) smoking. Beyond the particular contexts of smoking analyzed here, there is evidence that tobacco control activities may promote movement from daily smoking to intermittent smoking: states with more aggressive tobacco control policies have a greater proportion of ITS [14], which should reduce the burden of morbidity and mortality in those populations. Consistent with the idea that tobacco control policies promote a shift towards non-daily smoking, the prevalence of non-daily smoking in the US rose sharply between 1995 and 2001 [75] (though see [76]), a time of substantial tobacco control activity. The rise of non-daily smoking during this period is contrary to the expectation that declining prevalence rates would lead to “hardening” – that is, heavier smoking and increased dependence in the remaining population of smokers [77] – suggesting that tobacco control activity may lead to reduced dependence in some populations as well as reduced prevalence.

In summary, we showed that ITS' smoking was more strongly associated with environmental contexts and cues than DS'. Even where the smoking of both DS and ITS were associated with similar cues, the associations were stronger among ITS. Among ITS, who do not appear to have a need to maintain nicotine in the bloodstream, and thus have little or no dependence (as traditionally conceived) [54], triggering stimuli may play a dominant role in their documented difficulty quitting and widespread failure in cessation [21].
